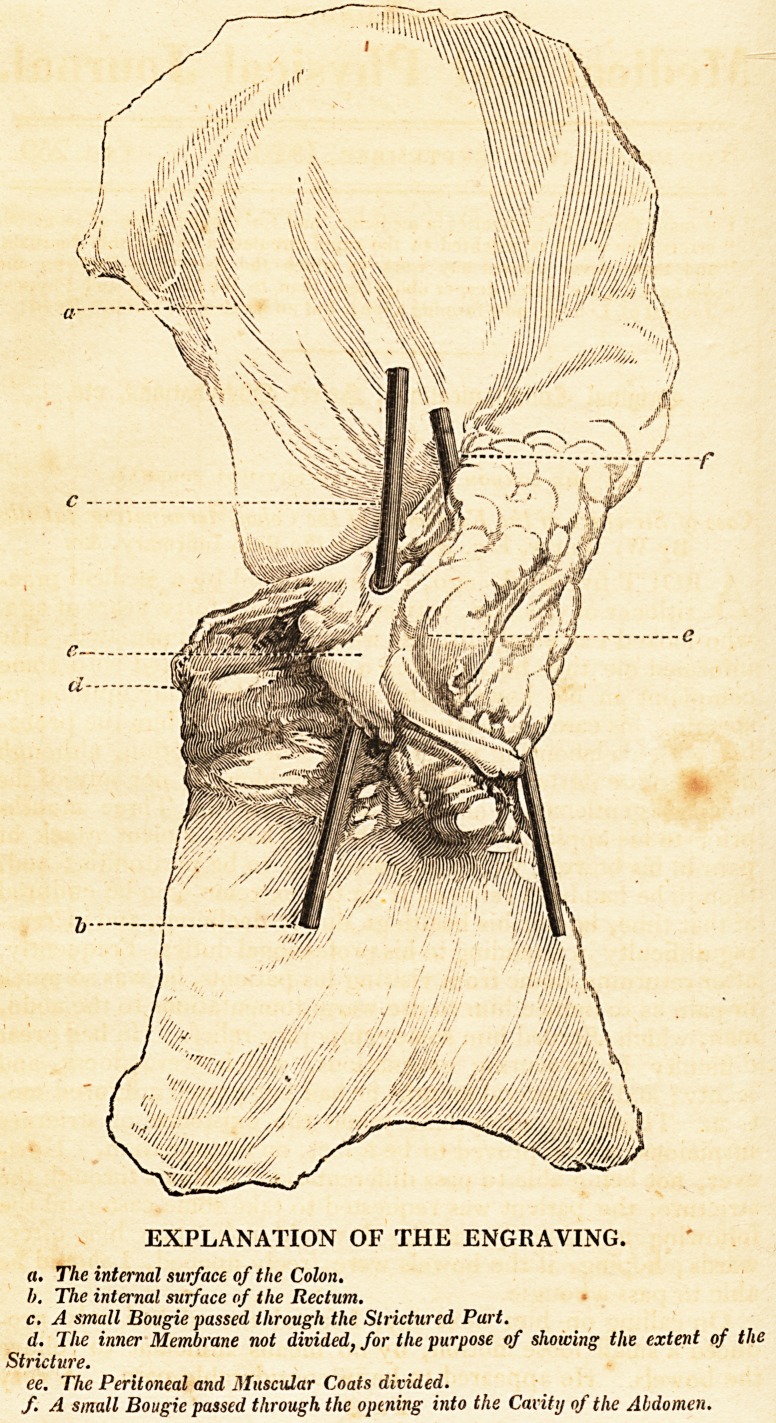# Case of Stricture of the Extremity of the Colon, Terminating Fatally

**Published:** 1820-09

**Authors:** W. White

**Affiliations:** Surgeon to the Bath Infirmary, &c.


THE LONDON
### Medical and Physical Journal.

3 OF VOL. XLIV.] SEPTEMBER, 1820. [NO. 259.

"For many fortunate discoveries in medicine, and for the detection of numerous "errors, the world is indebted to the rapid circulation of Monthly Journals; "and there never existed any work to which the Faculty in EUROPE and "AMERICA were under deeper obligations than to the Medical and Physical "Journal of London, now forming a long, but an invaluable, series." RUSH.

### Original Communications, Select Observations, etc.

FOR THE LONDON MEDICAL AND PHYSICAL JOURNAL.

### *Case of Stricture of the Extremity of the Colon, terminating fatally*

By W. WHITE, Esq. Surgeon to the Bath Infirmary, &c.

ABOUT five weeks ago, I was consulted by a medical practitioner of this city, between thirty and forty years of age, who looked extremely ill, and was very much emaciated. He informed me that he had been a long time afflicted with some complaint in his bowels, and wished to have my opinion respecting his case; it having been suggested to him the probability of his labouring under stricture of the rectum, although he had not entertained such an opinion himself, nor any of the medical gentlemen whom he had consulted. Three months prior to his application to me, he had had a violent attack of pain in his bowels, which he conceived to be peritonitis; and, though he had been relieved from the extreme pain he endured at that time, he felt his health gradually declining, and increasing difficulty in attending to his professional duties. Frequently, after returning home from visiting his patients, he was so much in pain as to induce him to use warm fomentations to the abdomen, which afforded him some temporary relief. He had great difficulty in procuring evacuations, which were loose and scanty; and for several months he had not passed a figured motion. These last symptoms made the existence of stricture suspicious, which proved to be a fact, on examination. However, not being able to pass different-sized bougies through the stricture, the patient was requested to take some castor-oil the following morning, promising I would call upon him afterwards; hoping, if the bowels were freely opened, I should be able to pass a bougie.

On calling on him next day, I found the oil had only produced a small loose motion, by no means sufficient to relieve the bowels. He appeared to be in considerable pain. A very small bougie was passed beyond the stricture, without meeting any other obstruction higher up the passage. A mixture with infusion of senna and sulphate of magnesia, was directed to be taken every two hours until the bowels should be freely open. In the evening, I found the mixture had not remained on the stomach, and that every thing he had taken returned. As the pain of the bowels had increased, I took about twelve ounces of blood from the arm, (which exhibited a buffy surface,) and he felt somewhat relieved afterwards. As aperients in a liquid form did not remain on the stomach, pills with extr. coloc. comp. &c. were directed to be taken at stated periods, until a proper effect should be produced.

The next morning, I found the stomach had rejected the pills also, and there had been no alvine evacuation. A very troublesome, and almost incessant, hiccough had come on; which, with the sickness and vomiting, continued two days, and then entirely ceased, in consequence of the patient having been directed to take some light curds and whey; the good effects of which I had often experienced in cases of obstinate vomiting. The patient was able again to keep down what nourishment he took, and also medicine. Various enemas were administered, and every means adopted that were likely to afford relief; but every attempt proved unavailing.* There was a total suppression of stools. The abdomen became distended, and the tumefaction continued to increase, with great languor and debility, until the morning of the eighth day (from my first seeing him), when he expired, after very severe suffering. It will appear obvious, from the dissection, that the effects of the disease had become of too serious a nature to admit of relief, after the discovery of the original cause.

The following were the appearances on dissection:

The tumefaction of the abdomen arose from a great quantity of flatus having escaped into the cavity, and a preternatural distension of the colon, particularly its ascending arch, which was of an enormous size, having more the appearance of the stomach than intestine. It was filled with soft frothy feces, which were beginning to escape into the cavity from two small openings at the upper part of the ascending arch, where the distention was the greatest. On tracing the intestine to the inferior extremity of the sigmoid flexure, a stricture was discovered, not more than sufficient to admit a very small bougie. The internal surface of the intestine had a healthy appearance, except that the coats above the stricture were very thin, from the long-continued distension they had been subject to: there

* The patient also had the advantage of Dr. Charles Parry's advice, and the kind assistance of other professional gentlemen.

was, however, a considerable thickening of the peritoneal and muscular coats at the stricture. Above the stricture there was a small opening into the cavity, but it did not appear that any feces had passed through it. There was no other appearance of disease.

On reading the Medical Journal of the present month, I was not a little surprised to meet with a paper, by a practitioner of this city, on stricture of the rectum; written, no doubt, with a view of claiming to himself the merit of discovering a complaint which has for many years been well known; and on which my Treatise, published first in 1812, has already reached a third edition.

Mr. Hicks says, "Towards the close of the year 1807, during my residence at Mr. Brookes's Theatre of Anatomy, some interesting cases of dissection came before me, which first suggested the probability of the intestinal canal being subject to simple contractions, without any apparent disease or disorganization." On referring to the Medical and Physical Journal of October 1809,* it appears, by the cases there reported, that I had met with the first of them five years previous to their publication; and that, by the summer of 1808, six cases of contracted rectum, under different modifications, had come within my particular notice; whilst, at that period, Mr. H. confesses to have met with only one case!—his first experiment with the bougie. Mr. H. also says, "My zeal for the promotion of that science which has been the object of my life, forbids me to withhold whatever information it is in my power to supply on this occasion." What information has he communicated? Nothing but what was known before. Surely, if in the course of eleven years numerous anomalous cases had been submitted to him, such an extensive practice must have supplied him with an ample opportunity of collecting materials for some important communication, long before the third edition of my "Observations on Stricture" appeared. Mr. H., however, concludes his paper by merely promising, "I may probably lay some of these interesting cases before the public at my first leisure."

If Mr. H. met with the first case of simple stricture as far back as the year 1807, it appears somewhat extraordinary that, so late as the year 1815, I was consulted by a person who had been under his care three months, without his having had any suspicion of the disease; at least, no examination with the bougie had been attempted, notwithstanding the symptoms of

---

* Vol. xxii.

† On a fair calculation, I should suppose that Mr. H. has had, for the last few years, five patients to my one, what were considered by him cases of stricture.

the complaint were very prominent. In the same year, also, I attended a gentleman with stricture of the rectum, and who had been a long time extremely hypochondriacal. By means of the bougie, the stricture gave way; but, as his mind continued in a very wretched state, I was informed that he afterwards consulted Mr. H.; who declared there was no stricture, but observed it was a fashionable disease with Mr. White. Here I may take the liberty of retorting on Mr. H. and say, that stricture has not only been a fashionable disease with him of late years, but, I have reason to believe, a very profitable one also!

With regard to Mr. H.'s knowledge of the disease itself, it does not appear, from the interesting cases of dissection he met with in the year 1807, that simple contraction actually existed in any of these cases; but that some unexplained circumstance only "suggested the probability of the intestinal canal being subject to simple contractions, without any apparent disease or disorganization." If simple stricture be not attended with some apparent disorganization, I am at a loss to comprehend how a local complaint could possibly be asertained; for, where there is no sensible deviation from the natural structure of a part, I should be glad to know by what other means simple constriction is to be detected, as all the cases of dissection I have hitherto met with of simple stricture of the rectum, have invariably been attended with more or less of structural derangement. It is evident to me that Mr. H. has not clear and distinct ideas on the subject, as he appears to confound permanent spasmodic stricture with simple stricture; and, although I have particularly described these different forms of constriction in the last edition of my "Observations on Stricture," (to which I have no doubt your readers will refer with satisfaction,) yet I have never met with a mere permanently spasmodic state of the rectum on dissection; and it is a matter of doubt if Mr. H. has had a single dissection of that nature since he left Mr. Brookes's dissecting-room.

With respect to his method of treating (what he calls) simple stricture, he relies, it seems, solely on the use of the bougie; and that "numerous anomalous cases, pronounced by practitioners of eminence to be purely idiopathic, and for a length of time ineffectually treated by them as such, but which, through the application of the bougie alone, have proved to be merely sympathetic." Mr. H. surely could not be ignorant of my having advanced this opinion eleven years ago in the Medical and Physical Journal; and subsequently, in each edition of my work on Stricture, additional cases have been produced in confirmation of it. Mr. H. does not inform your readers what kind of bougie he employs; and he carefully avoids noticing those invented by me, though they are preferred by all the medical gentlemen who have given them a trial to every other sort. I have however been informed, that he uses the elastic gum bougie, (and of a very large size,) which is certainly a very neat instrument; but I am persuaded, if it be relied on in making an examination where there is a suspicion of stricture, the practitioner will be liable to a deception, as the instrument, notwithstanding its great flexibility, does not readily yield at its extremity to the natural curvature of the passage; particularly should there be an unusual projection at the upper part of the sacrum, or last lumbar vertebra, the difficulty will be increased; which may lead an inexperienced surgeon to imagine there is a stricture, when in reality none exists.

I shall here take the liberty of mentioning a circumstance which occurred about four years ago, in the case of a gentleman, who, after having been a few weeks under my care with stricture of the rectum, could introduce my largest-size bougie with the greatest ease. As he intended shortly to sail for India, I thought it would be more eligible for him to use an elastic gum bougie, which he tried; and, though it was rather less in size than those he had been in the habit of using, he found it impracticable to introduce it. This result was a great disappointment, and matter of surprise to the gentleman; and still more so, when my attempt proved equally unsuccessful. He returned to the use of my bougies without any difficulty. The relation of the above fact may, perhaps, prove useful to the inexperienced and young practitioner.

*Bath; July 29, 1820.*

---

FOR THE LONDON MEDICAL AND PHYSICAL JOURNAL.

## Figures and Tables

**Figure f1:**